# Exploring PubMed as a reliable resource for scholarly communications services

**DOI:** 10.5195/jmla.2019.433

**Published:** 2019-01-01

**Authors:** Peace Ossom Williamson, Christian I. J. Minter

**Affiliations:** Director for Research Data Services, Libraries, University of Texas at Arlington, 702 Planetarium Place, Box 19497, Arlington, TX 76019, peace@uta.edu; Community Engagement and Health Literacy Librarian, McGoogan Library of Medicine, University of Nebraska Medical Center, 986705 Nebraska Medical Center, Omaha, NE 68198-6705, christian.minter@unmc.edu

## Abstract

**Objective:**

PubMed’s provision of MEDLINE and other National Library of Medicine (NLM) resources has made it one of the most widely accessible biomedical resources globally. The growth of PubMed Central (PMC) and public access mandates have affected PubMed’s composition. The authors tested recent claims that content in PMC is of low quality and affects PubMed’s reliability, while exploring PubMed’s role in the current scholarly communications landscape.

**Methods:**

The percentage of MEDLINE-indexed records was assessed in PubMed and various subsets of records from PMC. Data were retrieved via the National Center for Biotechnology Information (NCBI) interface, and follow-up interviews with a PMC external reviewer and staff at NLM were conducted.

**Results:**

Almost all PubMed content (91%) is indexed in MEDLINE; however, since the launch of PMC, the percentage of PubMed records indexed in MEDLINE has slowly decreased. This trend is the result of an increase in PMC content from journals that are not indexed in MEDLINE and not a result of author manuscripts submitted to PMC in compliance with public access policies. Author manuscripts in PMC continue to be published in MEDLINE-indexed journals at a high rate (85%). The interviewees clarified the difference between the sources, with MEDLINE serving as a highly selective index of journals in biomedical literature and PMC serving as an open archive of quality biomedical and life sciences literature and a repository of funded research.

**Conclusion:**

The differing scopes of PMC and MEDLINE will likely continue to affect their overlap; however, quality control exists in the maintenance and facilitation of both resources, and funding from major grantors is a major component of quality assurance in PMC.

## INTRODUCTION

The National Library of Medicine (NLM) creates and maintains resources that are at the heart of library services relating to health information. NLM’s mission has always included a focus on supporting health care research and practice and providing access to trustworthy and timely health information [1]. When NLM expanded its reach through online services, its feature product, MEDLINE, continued in that tradition. PubMed delivers a publicly available search interface for MEDLINE as well as other NLM resources, making it the premier source for biomedical literature and one of the most widely accessible resources in the world. Health sciences practitioners, researchers, faculty, and students have repeatedly reported PubMed and MEDLINE as one of the few sources they use to search literature [2–5].

As research, publishing, and access to scholarly resources have evolved over recent years, it is important to determine the role that PubMed and other NLM resources play in the dissemination of research and other scholarly output. Librarians are increasingly expected to assist with research and publishing issues, including ensuring funding compliance, conducting literature reviews, navigating open access, understanding copyright, measuring impact, working with data, and disseminating research [6, 7]. As a result, medical librarians and researchers depend on NLM resources as trustworthy sources of quality literature.

### About PubMed, MEDLINE, and PubMed Central

For many users, PubMed is synonymous with the MEDLINE database. In 1971, NLM created MEDLINE to serve as the online version of the Medical Literature Analysis and Retrieval System (MEDLARS). MEDLINE (or MEDLARS online) consists of life sciences and biomedical journal citations that are indexed with NLM Medical Subject Headings (MeSH) [8]. Originally, the MEDLINE service could only support up to twenty-five users simultaneously, and access was available primarily in medical libraries [9]. To improve the availability of MEDLINE, NLM released the PubMed search engine as part of the Entrez retrieval system, beginning as an experimental database in 1996 [10].

As of June 1997, PubMed provides free and unlimited access for all users through the Internet [11]. Over time, PubMed became more than a public interface for MEDLINE citations and publisher links to full-text: it has undergone numerous transformations to improve usability and functionality through several redesigns, and introduced features including LinkOut and Single Citation Matcher [12]. PubMed has also provided a pathway toward increased accessibility to research by expanding to include access to PubMed Central and the National Center for Biotechnology Information (NCBI) Bookshelf [13–15].

**Figure 1:**
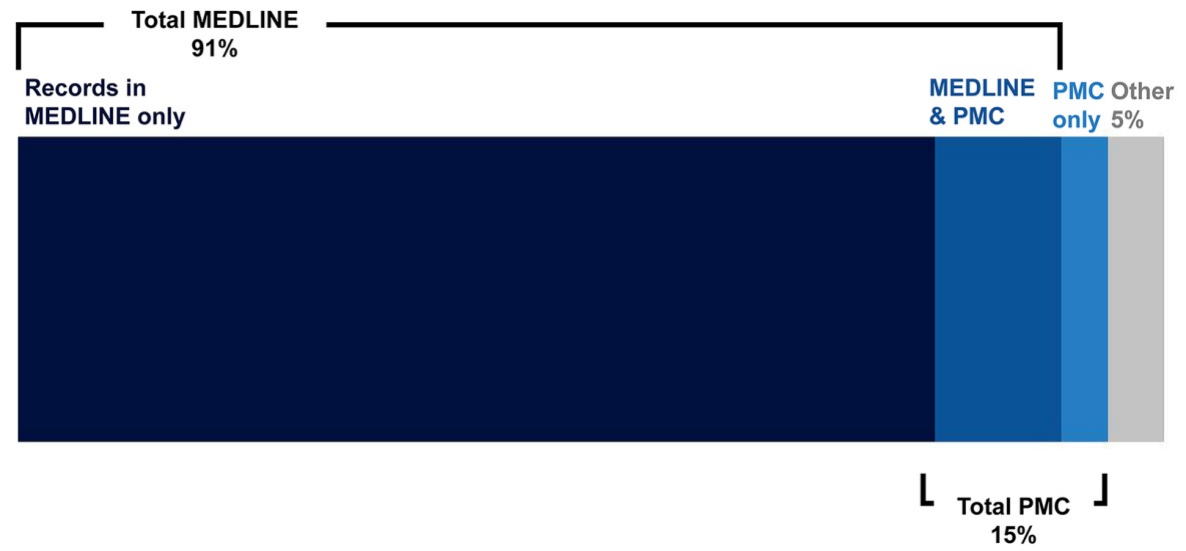
Composition of PubMed in 2017

As of October 2017, PubMed contained 27.5 million records, representing approximately 7,000 journals [16]. Together, records from PubMed Central (PMC) and records included in or marked for inclusion in MEDLINE make up almost 95% of PubMed (Figure 1). Bookshelf is approximately 1% of PubMed, and the remaining 4% consists of several types of records, including out-of-scope citations from MEDLINE journals, citations that precede the date that a journal is selected for MEDLINE, and new records submitted by publishers that have not yet been reviewed by NLM staff.

The largest percentage of records in PubMed comes from MEDLINE, and the Literature Selection Technical Review Committee (LSTRC) is responsible for reviewing and recommending journal titles to include in MEDLINE. LSTRC assesses the scope, quality of content, accessibility of foreign-language articles, technical quality, and publishing practices. Also, “journals must be able to submit XML tagged data, and electronic-only journals must provide robust current access to all of its content and have an acceptable arrangement for permanent preservation of, and access to, the content” [17]. Subject expertise and relevance are additional criteria. At least 20% of content must relate to biomedicine and health, and journals that are accepted are oriented toward original research and provide the highest expertise in the field. Rejected journals can reapply after 2 years. If rejected after a second submission, journals can then reapply every 3 years [16–18]. As of October 2017, MEDLINE contained 25 million records from over 5,600 journals [16].

The second largest component of PubMed is PMC. Launched in 2000, PMC serves as a permanent digital archive of full-text life sciences and biomedical journal articles. PMC also includes articles deposited by journal publishers and “author manuscripts,” in other words, published articles that are submitted in compliance with the public access policies of the National Institutes of Health (NIH) and other research funding agencies [15, 19–21]. NLM provides public access to the contents of PMC and manages a collaborative called PMC International to store copies of its contents in local deposits at centers in multiple global locations [15, 22]. Publishers supply 88% of the content in PMC through active provision of current or historical content or through selective deposits, and the remaining 12% of PMC comes from author manuscripts. As of October 2017, PMC contained 4.5 million articles.

**Figure 2:**
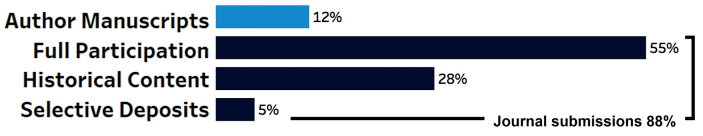
Composition of PubMed Central in 2017

Publishers submit an application and sign an agreement to participate in PMC, and there are different options for contributing content (Figure 2). Journals that agree to *full participation* deposit their entire issues in the archive on an ongoing basis and account for more than half of PMC. Scanned *historical content* represents 28% of the content and includes back issues of biomedical journals that NLM has identified as having historical significance. *Selective deposits* account for 5% of the content and include open access articles from hybrid publishers and articles deposited to support specific funding agency policies. As of May 2018, selective deposit is limited to journals indexed in MEDLINE [16, 17, 23].

NLM staff review journals prior to including them to determine if they meet the criteria of the *Collection Development Manual,* which specifies the aim to acquire primarily scholarly literature “pertaining to health care, to the practice of the science and art of medicine broadly conceived, and to those branches of the life sciences which are fundamental to that science and art” [15, 24]. An external panel of independent experts assesses the journal’s scientific, editorial, and technical quality, and the NLM Library Operations Division makes the final decision. This process was implemented in 2014 following the approval of the PMC National Advisory Committee because of a significant increase in journals applying to participate. Rejected journals can reapply after two years [16, 25, 26].

PMC is also the designated repository for twelve US agencies and organizations and twenty-seven European funders [16, 21]. Author manuscripts are deposited in compliance with these and other funders’ public access policies. The policies require that literature resulting from specified funded research must be made available in PMC within six to twelve months of publication, depending on the funder policy [19, 20]. The manuscripts are distinguishable from other content by the author manuscript banner and runner down the left side of the page.

Other NLM resources that may be associated with PubMed are the NLM Catalog, PubMed Health, and MedlinePlus. The NLM Catalog contains bibliographic records for over 1.4 million journals, books, audiovisuals, electronic resources, and other materials. It also includes detailed indexing information for journals in PubMed and other NCBI databases, but not all materials in the NLM Catalog are part of NLM’s collection [16]. While PubMed Health and MedlinePlus have similar names to the aforementioned resources, these resources are built for different uses: PubMed Health provides reviews of clinical effectiveness research for health care providers and patients and will be retired in 2018, and MedlinePlus is a consumer health website providing information on various health topics, drugs, dietary supplements, and health tools [27, 28].

### Scholarly communication uses of PubMed, MEDLINE, and PubMed Central

As librarians respond to the growing demand for knowledge and expertise about publishing, author rights, and access, PubMed has served as a resource for supporting these services. Authors are looking at options for open and public access to their research, and a 2013 memorandum from the US Office of Science and Technology Policy (OSTP) provided greater motivation for increasing research availability. The OSTP memorandum instructed federal agencies with more than $100 million in annual research expenditures to consult with stakeholders and implement a plan for public access within 6 months [29]. Thus, many authors must now comply with open and public access mandates from publishers and funders, many of which resulted from the 2013 OSTP memorandum.

Navigating the publishing process through the proliferation of predatory publishers adds another challenge for authors [30]. Authors are increasingly turning to libraries for information that will guide them through the research and publishing process. Advisory organizations are also recommending the library as a resource. For example, the Federal Trade Commission (FTC) recommends checking with a librarian before submitting an article to a journal to avoid predatory publishers [31]. NIH supported the FTC’s recommendations in a November 2017 notice that provided advice for authors who are publishing the results of NIH-funded research. This statement provides recommendations for identifying credible journals and implores its stakeholders (including librarians) to help authors engage in effective scholarly communication practices. NIH also recognizes the role of NLM in maintaining PubMed and PMC and encouraging publishers to follow established industry best practices [32].

Publishers, librarians, and authors rely on PubMed/MEDLINE, among other resources, as a vetting tool. For publishers, having a journal indexed in MEDLINE means that it has met stringent criteria for quality—which attracts potential authors, subscribers, and readers—and journals receive more submissions after their acceptance into MEDLINE. Publishers also value the MeSH metadata and the increased discoverability through PubMed or private vendors that provide access [21]. MEDLINE is an important tool for librarians to help users find trustworthy journals in which to publish. In addition, authors who publish in MEDLINE-indexed journals can often reach a larger audience due to health professionals’ and medical librarians’ preference for PubMed and MEDLINE to search for literature.

PMC also contributes to scholarly communication efforts because it provides public access to research. Funders support this archive because research resulting from public funding should be available to the public [29, 33]. Providing easier access also helps to translate research into practice. Evidence-based practice can be inhibited when practitioners are unable to access research behind paywalls. Public and open access can also improve the likelihood of an author’s work being cited and can benefit the teaching, collaboration, and implementation of research [29, 34–36]. Materials do not have to be open access to be included in PMC, and most materials that are currently available are under copyright. Librarians and researchers using materials found in the archive must still comply with the articles’ copyright and license terms.

There has been some concern about the quality of PubMed content from sources other than MEDLINE. Some of this concern comes from a misunderstanding of the different components of PubMed. That is, although comparisons are often made between PubMed and MEDLINE as if they are two different databases, MEDLINE is in fact a subset of PubMed. There has also been misrepresentation that PubMed is an index itself [37, 38]. The main focus of this concern centers on the inclusion of journals in PubMed that were identified by Jeffrey Beall on his list of “potentially predatory publishers,” which is no longer updated and is archived at beallslist.weebly.com.

Other concern centers on PMC as affecting PubMed’s quality, particularly author manuscripts that are deposited into PMC and automatically included in PubMed without any review process. There are also criticisms that the PMC inclusion criteria for participating journals are less stringent than MEDLINE inclusion criteria [39, 40], although author manuscripts are peer-reviewed manuscripts that have been accepted for publication and deposited in compliance with a funder’s policy; some refer to this as the “PMC backdoor.” One author states, “PubMed’s brand has long been muddled in ways that pass lower-quality works through the system under cover of prestige. This has real consequences” [39]. This argument exists largely on the assumption that lower-quality publications—as opposed to journals reviewed and included in MEDLINE—are increasingly found in PubMed due to NIH-funded research published in journals managed by predatory publishers.

The authors did not investigate the first concern regarding the percentage of “predatory publishers” in PubMed because it centers on a list that is highly disputed as reputable [41]. Furthermore, publishing in a potentially predatory publication does not automatically equate to poor scientific methods in an article, and when articles from publications in Beall’s List were assessed recently, PMC and PubMed were found to have lower numbers of these articles than resources like Scopus and Google Scholar [42].

Our aim was to examine whether there has been a change in the proportion of PubMed content indexed in MEDLINE, and if so, whether PMC is contributing to this shift. We also examined whether the deposit of author manuscripts provides a “PMC backdoor” for low-quality research. In addition, we interviewed representatives speaking on behalf of NLM and an expert consultant for the PMC journal review process to provide context regarding the history, purpose, and quality control of these resources.

## METHODS

We collected data in November 2017 through searches via the PubMed interface along with calculations in Excel. Data were obtained and organized according to the year that records were created in PubMed to show the trend in items added to PubMed each year. Data were not organized by publication year because records are not always added to PubMed in the same year that they are published. PMC records were also retrieved by searching PubMed using the subset *pubmed pmc[sb]*, which retrieves live or available journal article records in PMC (i.e., excluding articles currently under embargo).

Because PubMed provides a public access interface for MEDLINE, we investigated current practices relating to the addition of records in PubMed to examine whether MEDLINE-indexed articles continue to represent the majority of PubMed records. To do this, we obtained the number of new records added to PubMed in 1990 and to both PubMed and PMC for the years 2000 to 2017. We also obtained the number of new records that were indexed in MEDLINE and still in-process to be indexed in MEDLINE during the same time span in order to determine the ongoing percentage of records in each resource that is or will be indexed in MEDLINE.

For the purpose of our analysis, MEDLINE records included the records in PubMed marked as either *medline* or *inprocess* in the subset field. The subset field retrieves records by citation status, subject, or journal category, with the search tag *[SB]* [43]. The records marked as *medline* have been indexed with MeSH and, if relevant, may be linked to the NCBI Gene database or included in Supplementary Concept Records for substances that are not in MeSH [44, 45]. The in-process records have been identified for inclusion in MEDLINE, but the indexing process is not completed yet; therefore, any records with the *inprocess* subset would be missed if a user searches PubMed using only MeSH (or any MEDLINE-only interface).

Data were collected on the number of author manuscript records in PMC from 2005 to 2017 along with the number of these records that were also indexed in MEDLINE to determine the percentage of PMC content that was author manuscripts and the percentage of author manuscripts that was indexed in MEDLINE in recent years. All trends were compared prior to and following the NIH public access mandate to determine whether the mandate served as a motivation for authors to deposit their articles and for journals to participate in PMC to attract potential authors’ submissions.

Using an adaptation of the Comparing Means and Proportions spreadsheet created by Princeton Data and Statistical Services, we performed descriptive analysis of trends via *z*-test across years, with a significance threshold of *a*=0.001. Supplementary calculations were made in Tableau Desktop 10.1.1 to create figures. Supplemental Appendix A provides the formulas we used to perform the statistical analyses.

We also interviewed Joyce E. B. Backus, NLM associate director for library operations; Kathryn Funk, NLM program manager for PMC; and Laurey Steinke, an expert consultant for the PMC journal review process and assistant professor at the University of Nebraska Medical Center Department of Biochemistry and Molecular Biology, for additional qualitative details regarding PubMed, MEDLINE, and PMC. Deborah Ozga, NLM head of the Index Section, and Rebecca Stanger, NLM journal publisher liaison, provided additional information via email.

## RESULTS

**Figure 3:**
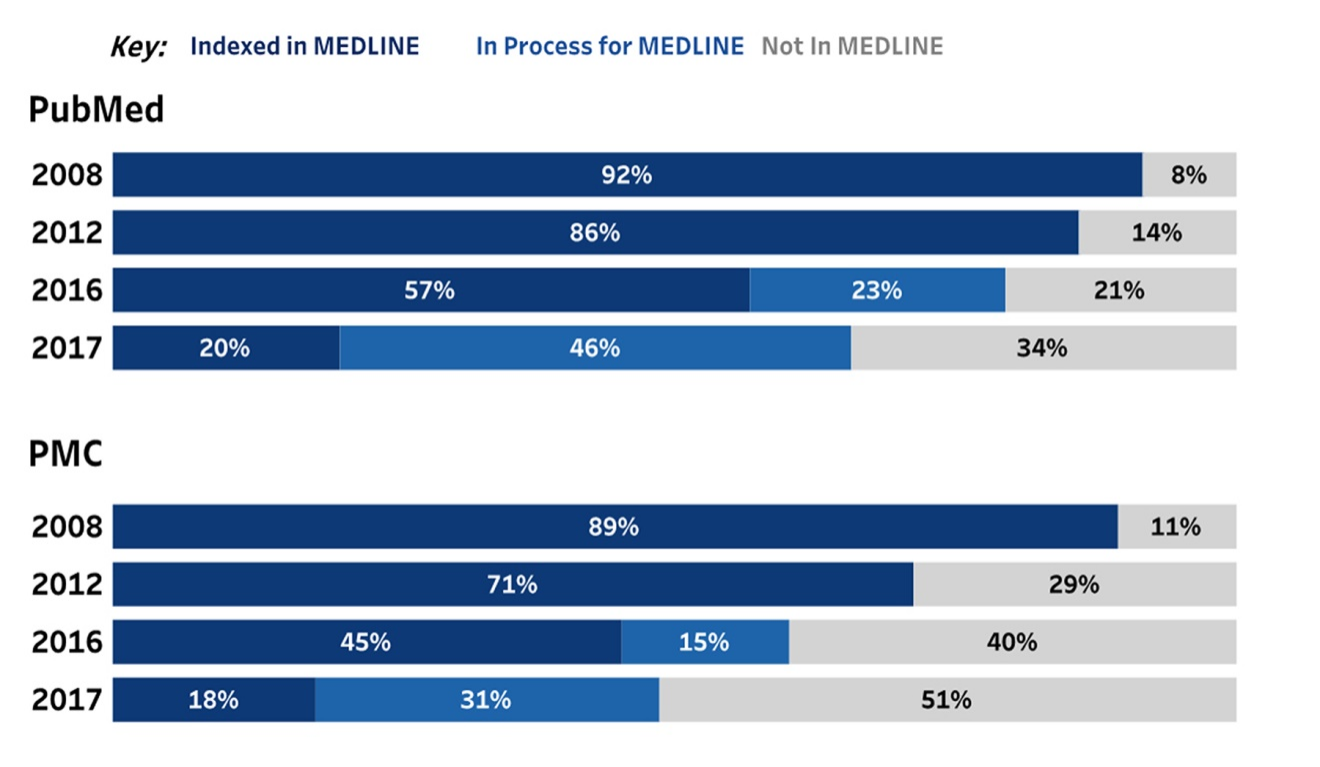
MEDLINE-indexing of new records in PubMed and PMC by year

PubMed remains primarily composed of MEDLINE records, but this composition has changed slowly over time. We can see this in the past decade: 96% of PubMed consisted of MEDLINE records in 2008, whereas 91% of PubMed consisted of MEDLINE records (including in-process records) in 2017. The cause of the overall composition shift was evident when we investigated the records that were added each year to determine the percentage that were indexed in MEDLINE, in process to be indexed in MEDLINE, and not indexed in MEDLINE. In both PubMed and PMC, records outside of MEDLINE composed a larger percentage of new records each year (Figure 3).

### PubMed’s growth and backlog

**Figure 4:**
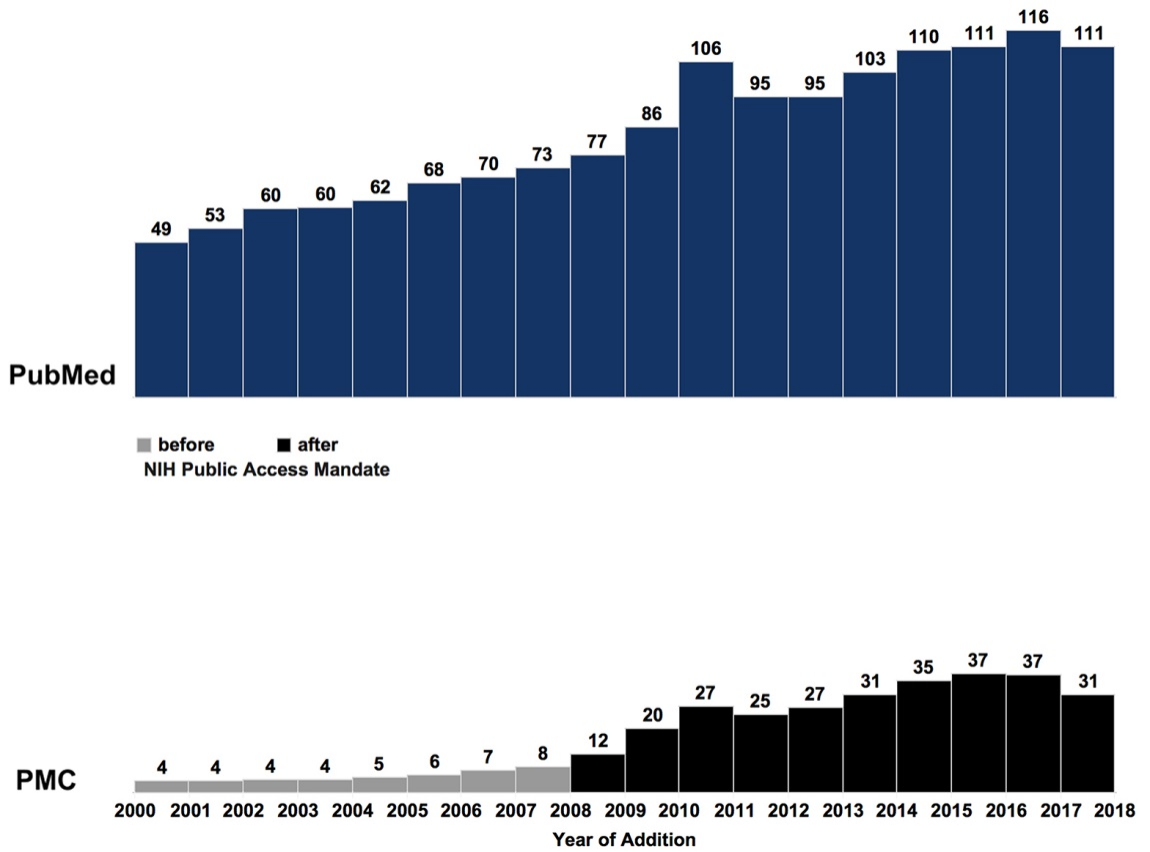
New PubMed and PMC records added by year (by 10,000s)

The number of records added to PubMed and PMC increased between 2000 and 2017 (Figure 4). Not surprisingly, there was a jump in new PMC records following the 2008 NIH public access mandate.

In November 2017, over 800,000 new PubMed records that had been created between 2011 to 2017 were still in-process and not yet indexed in MEDLINE. More than 265,000 and 506,000 of these new records were created in 2016 and 2017, respectively. In her email correspondence, Ozga acknowledged a backlog and mentioned that NLM is working toward developing variations of the Medical Text Indexer (MTI) algorithm, such as the MTI First Line Indexing, for semi-automated or fully automated indexing to reduce the backlog of in-process records [13, 34].

### MEDLINE representation in PubMed

**Figure 5:**
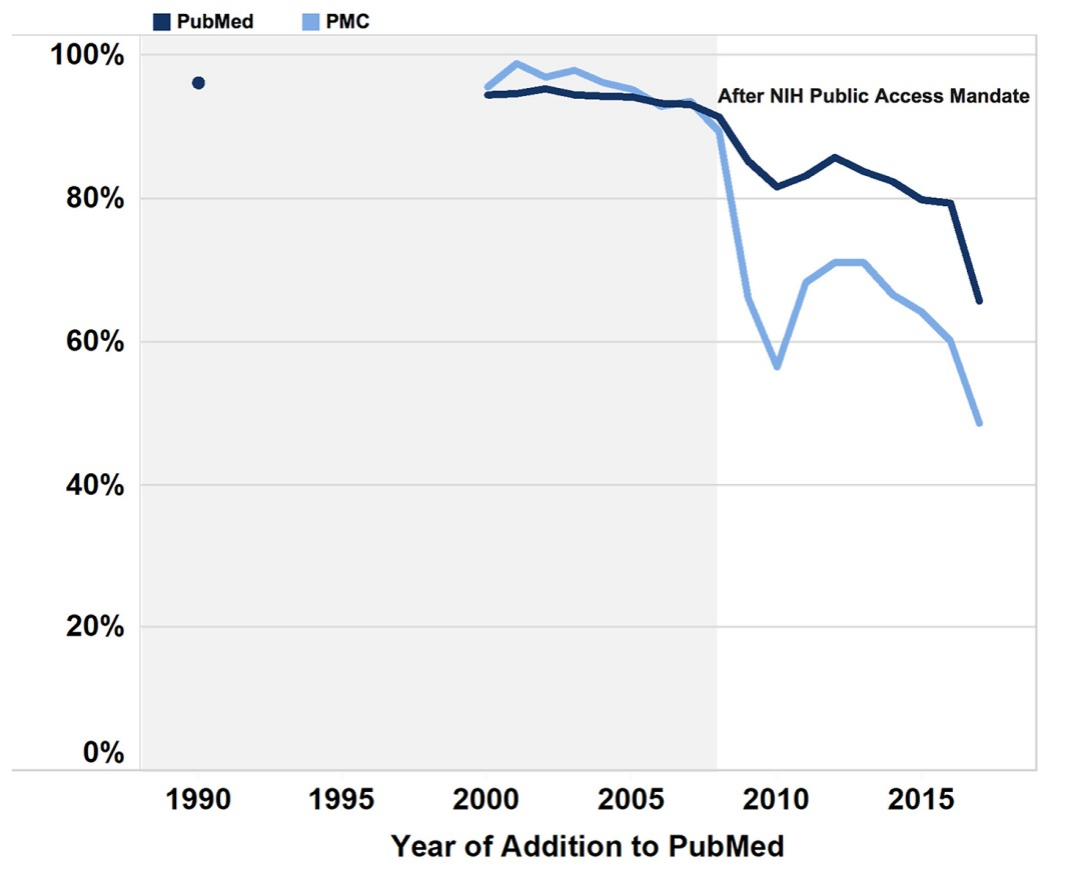
Percentage of new PubMed and PMC records indexed in MEDLINE

We obtained the number of new records added to PubMed in 1990 and to both PubMed and PMC for the years 2000 to 2017. Although the proportion of new PubMed and PMC records that were indexed in MEDLINE varied across years, a pronounced change in the proportion of MEDLINE-indexed PMC records was observed after the NIH public access mandate went into effect in 2008, whereas the trend in PubMed records was similar but less extreme (Figure 5). It is important to note that PMC records are a subset of PubMed records.

Bonferroni-adjusted *p*-values showed that the proportion of MEDLINE-indexed records differed significantly between PubMed and PMC (*p*<0.001). We also tested the proportion of new PubMed and PMC records across years to identify trends in their MEDLINE indexing. New PubMed records were compared between 1990 (10 years before PMC), 2000 (at PMC’s start), and 2008–2017 (in the past decade). New PMC records were compared between 2000 and 2008–2017. The proportion of new MEDLINE-indexed PubMed records (out of total records) differed significantly across all year comparisons (*p<*0.001), except for between 2010 and 2014 (*p*>0.001). The proportion of new MEDLINE-indexed PMC records also differed significantly across years (*p<*0.001), except for between 2009 and 2014 (*p>*0.001) and between 2012 and 2013 (*p>*0.001).

**Figure 6:**
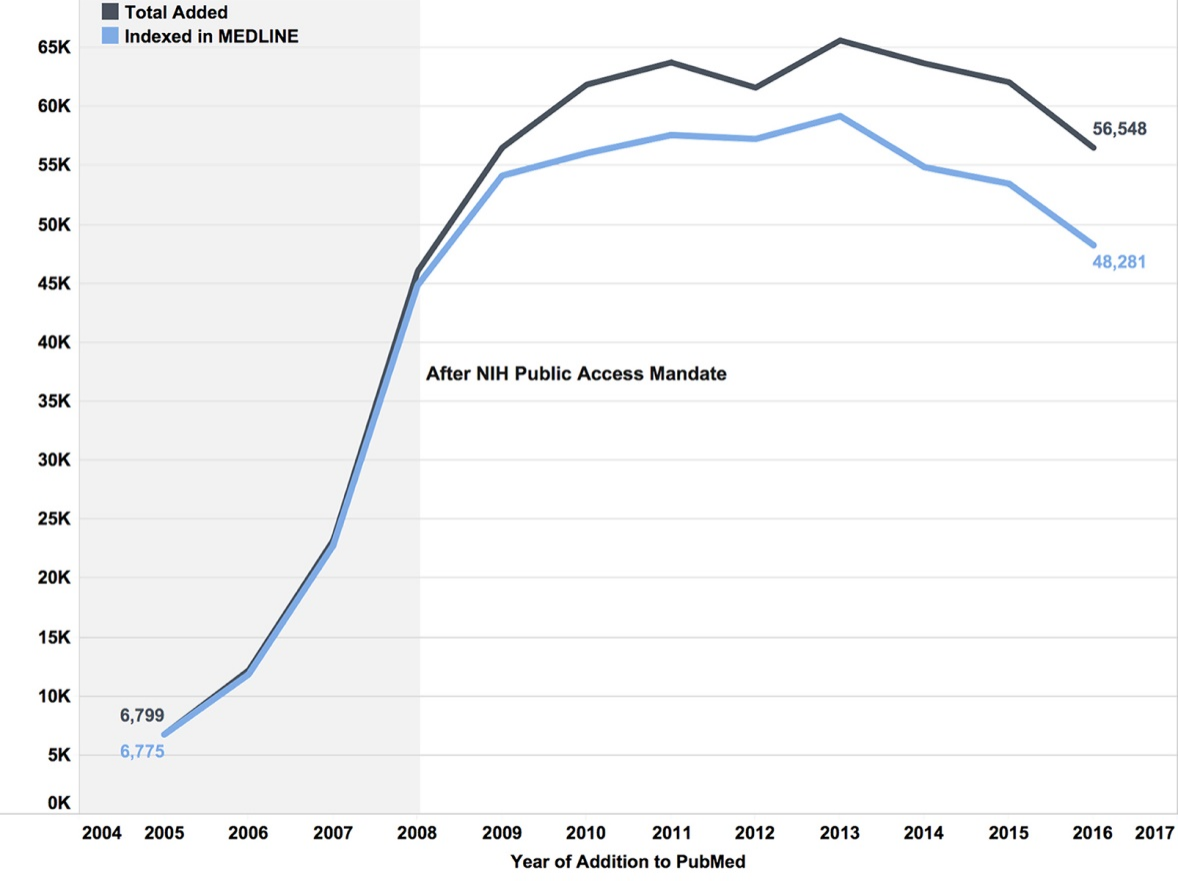
New author manuscript records added to PubMed

We next examined the number of new records for author manuscripts in PMC from 2005 to 2017 to look at trends of publication in MEDLINE journals. Before the NIH public access mandate in 2008, almost all author manuscripts in PMC were published in MEDLINE-indexed journals. From 2005 to 2011, author manuscripts were submitted at an increasing rate, and, as of 2017, more than two-thirds of these records were still published in MEDLINE-indexed journals (Figure 6). Although they are called “author manuscripts,” Funk stated that over 70% of the manuscript submitted to the NIH Manuscript Submission System (NIHMS) were publisher-initiated to help authors comply with the NIH public access policy [25]. Even though more manuscripts were being deposited to comply with public access policies, these submissions were still only 12% of PMC and were an even smaller component of PubMed (<2%).

While funding mandates dictate deposit and PMC author manuscripts are automatically included in PubMed, NLM staff added by email that “NIH and other funders do not dictate the journals in which their funded authors may publish. Consequently, author manuscripts in PMC may be from journals that have not yet undergone scientific review by NLM, are traditionally out of scope for the NLM collection, or have not met NLM’s standards for PMC” [17].

**Figure 7:**
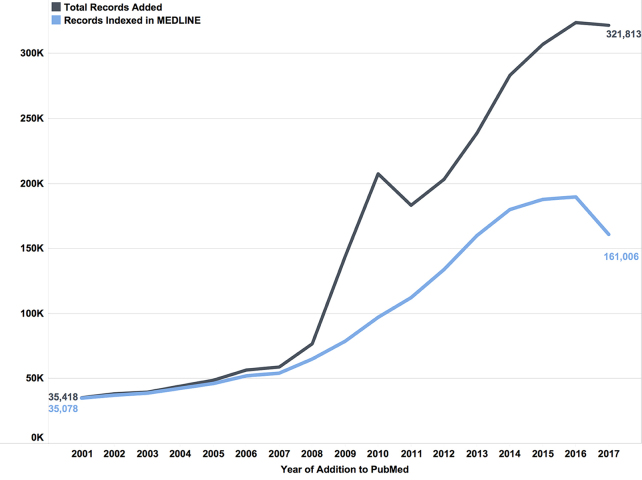
PubMed records added from PMC publisher submissions Note: This chart displays records from participating journals’ contents and from scanned historical materials. It does not include records from author manuscripts that were submitted by the publisher.

We also examined the number of PMC records from publishers that deposited either entire issues through full participation or selective deposit of materials that are not related to compliance with public access policies. This represents approximately 83% of PMC and has undergone the largest change in MEDLINE indexing over time. We excluded author manuscript records from this search. After the NIH public access mandate took effect in 2008, the number of new publisher-submitted records added to PMC doubled, and the number of new publisher-submitted, MEDLINE-indexed records dropped by half (Figure 7).

When asked about the changing percentage of new records indexed in MEDLINE, Backus stated that although NLM’s aim is not to maintain a certain proportion of MEDLINE records in PubMed or PMC, there is hope that more MEDLINE-indexed journals will be deposited in PMC for long-term preservation and broader access [18]. Backus also noted that it is important to understand the different aims of MEDLINE and PMC, as established by NLM. The aim of MEDLINE is to provide a highly selective index of journals in biomedical literature. Conversely, PMC’s aim is to provide a permanent archive for good-quality research, so any journal that meets NLM’s standards for scientific and editorial quality will be accepted [8, 26]. Publishing industry best practices are considered for both MEDLINE and PMC [24].

In response to criticisms of the increase in non-biomedical content deposited in PMC, both Funk and Backus emphasized that PMC provides a vehicle for research efficiency and broader content, while supporting public contributions to scientific research. Numerous funders list PMC as their mandated archive, including the Bill and Melinda Gates Foundation and all Department of Health and Human Services operating divisions (e.g., Centers for Disease Control and Prevention, Food and Drug Administration, and Centers for Medicare and Medicaid Services) [33]. The Department of Homeland Security began using PMC as an archive in 2018 [25]. Funk stated that including this non-biomedical content has value: some people might not easily find the literature if it is not included in the archive, and it is helpful for science to be less siloed, as there is some overlap between research domains [25]. For example, research occurring on the space station is beneficial to the biomedical community’s understanding of different aspects of human health [25].

### Ongoing quality control

In the interviews, Steinke, Backus, and Funk mentioned the reevaluation processes that occur for MEDLINE and PMC. Sometimes poor-quality journals exist in both resources because no process is perfect. Also, the quality of journals can change over time, reflecting changes in editorial leadership or publishers. Reviews have occurred for MEDLINE since LSTRC was formed in 1988, but these reviews typically centered on a specific topic. For example, the American Hospital Association and American Dental Association recommended journals in specific areas. This topic-centered approach had not occurred in recent years, but a publication that has been included in MEDLINE can be reviewed if issues are noted with publication quality, production problems, or nonconformance with industry best practices [25, 26].

PMC has had an informal reevaluation process for years, but a more formal process was implemented in 2017 [25]. PMC journal managers perform ongoing quality assessment of features like the volume of content produced by journals and changes in journal practices. PMC staff also keep up with user reports of systemic problems and comments that are made about a resource through online conversations [25]. If there are verifiable concerns about the scientific or editorial quality of the content in a PMC journal or significant changes in its ownership, policies, or practices, a journal may undergo another review. Before a reevaluation begins, NLM staff will notify the journal of the concerns and place a hold on processing new content during the review. The reevaluation process is similar to the review process for new journal applications, including evaluation by external consultants, and the decision to continue or discontinue archiving journal content in PMC is final [17].

## DISCUSSION

The diminishing percentage of MEDLINE-indexed records in PubMed is likely due to PMC as a growing component of PubMed. There are criticisms of PMC’s inclusion in PubMed, referring to it as a backdoor option for literature that is not indexed in MEDLINE. This “PMC backdoor” is blamed for reducing the quality of PubMed. In a blog post, Michelle Kraft, AHIP, former president of the Medical Library Association, compared PMC manuscripts appearing in PubMed to medical advice from Gwyneth Paltrow’s Goop site being published on WebMD [40].

However, author manuscripts are currently the smallest percentage of PMC content (12%) and an even smaller component of PubMed (<2%). In addition, most manuscripts deposited by authors in PMC are published in MEDLINE-indexed journals, and this rate has remained high over time. Because the majority of manuscripts deposited by authors in PMC come from MEDLINE-indexed journals, it could be argued that the funding agencies’ grant review process provides some quality control for author manuscripts in PMC. Funk also stated a similar claim in the interview [25]. However, we reiterate that inclusion in MEDLINE does not guarantee good scientific methods to users searching for research. Likewise, authors should still critically assess publication venues and make good choices in determining where to publish, especially when they submit articles from funded research, because predatory publishers can make readers question research results.

In regard to journal submissions, the rate of additions coming from publishers is increasing, likely due to an interest in participating in the movement toward more open research that is reflected by authors’ publishing habits and public access mandates. However, any journal that is not indexed in MEDLINE with a formal agreement to deposit in PMC must go through a review process first. This review process has become more stringent in recent years. There is also a formal reevaluation process to address problem journals that have already been accepted.

As the scholarly environment continues to change and research is available through interfaces that interact with thousands of journals, it is important to evaluate an article on its own merit in addition to evaluating the publication as a whole. This issue is not unique to PubMed, with researchers having previously found some potentially predatory journal titles in other databases, including the Directory of Open Access Journals and Academic Search Complete [46–48].

Librarians advise users on distinguishing quality research through critical appraisal and assist authors in searching for and choosing reputable publishers. Most use a number of tools in this process. NLM encourages users to visit the NLM Catalog for additional information about journals in PubMed, including their indexing status (MEDLINE or non-MEDLINE), their selection for the NLM collection, and their participation in PMC [16, 49]. In addition, NIH offers recommendations for resources to evaluate journals, such as using Think Check Submit and becoming familiar with publishing best practices [32]. Multiple publications also explain how to critically read and evaluate the quality of scientific research at the article level [50–52].

### Distinguishing resources in PubMed

In regard to PubMed and the various resources it encompasses, it would be beneficial for users if NLM provided a clearer indication from which particular resources a record stems. It can be confusing to navigate the different types of content in PubMed, especially because there is so much overlap. Many people view a journal’s inclusion in MEDLINE as a credential that weighs in a journal’s favor because of the LSTRC review methods, even though PMC journals are also evaluated for scientific quality. Users still want the ability to filter results based on whether records are indexed in MEDLINE. Currently, the status of the different record types in PubMed is somewhat buried in the search results. There are no visible tags for other statuses (e.g., in process or publisher-supplied) available on the search results page, and the status tag [Indexed for MEDLINE] is only visible in the abstract view. Previously, this status was available in the summary view.

There are multiple ways to search PubMed for MEDLINE-only citations, including by using solely MeSH terms in a search strategy, using the MEDLINE filter on the search results page, adding the *medline[sb]* tag to a search strategy, or searching MEDLINE through a licensed vendor interface. However, limiting a search to only MEDLINE records often excludes the most current research, as many articles are still in-process due to the indexing backlog. As NLM develops a solution for the backlog, it may become easier to search MEDLINE, but it is crucial for users to note that PMC also includes quality research and is providing increasing public access to literature that might otherwise be behind paywalls.

### Limitations

Our findings are only as accurate as the retrieval of results using the NCBI PubMed interface, and the numbers reflect what was displayed in the results of search strategies that we developed and that NLM did not validate. There was also a discrepancy between totals when searching *all[sb]* in PMC and *pubmed pmc[sb]* or *pubmed pmc all[sb]* in PubMed, likely due to a small collection of PMC records that were not included in PubMed. It is also possible that some records were added to PubMed through MEDLINE before the full text was deposited in PMC in a different year. In addition, some PubMed records are still in the “publisher supplied” status, which means that they were recently added by the publisher, but NLM staff have not yet distinguished whether they will eventually be indexed in MEDLINE.

## CONCLUSION

The percentage of MEDLINE records in PubMed has been slowly decreasing; however, whether that trend will continue and the meaning and effect of this shift is not clear. Further research is necessary to investigate the impact of the increase in PMC content, especially the impact of the new review policies and the contributions of journals that fully participate, on the role of PubMed for users who are searching for literature and for authors who are attempting to seek validation for publications in which to publish. In addition, there is a lack of studies investigating the research quality of literature retrieved through PubMed as well as other resources, using proven critical appraisal methods rather than comparisons with lists of journals and publishers, like those created by Beall. Research of this caliber will support librarians’ efforts to encourage users to engage in the same types of evaluations when searching for literature and choosing where to submit research articles.

## SUPPLEMENTAL FILE

Appendix Formulas used by the authors to perform their statistical analyses
